# Individual differences in the neural dynamics of visual narrative comprehension: The effects of proficiency and age of acquisition

**DOI:** 10.3758/s13423-023-02334-x

**Published:** 2023-08-14

**Authors:** Emily L. Coderre, Neil Cohn

**Affiliations:** 1 Department of Communication Sciences and Disorders, University of Vermont, 489 Main St, Burlington, VT 05405 USA; 2 Department of Communication and Cognition, Tilburg School of Humanities and Digital Sciences, Tilburg Center for Cognition and Communication (TiCC), Tilburg University, Tilburg, The Netherlands

**Keywords:** Visual narratives, Visual language, Event-related potentials, Proficiency, Age of acquisition, Comics

## Abstract

Understanding visual narrative sequences, as found in comics, is known to recruit similar cognitive mechanisms to verbal language. As measured by event-related potentials (ERPs), these manifest as initial negativities (N400, LAN) and subsequent positivities (P600). While these components are thought to index discrete processing stages, they differentially arise across participants for any given stimulus. In language contexts, proficiency modulates brain responses, with smaller N400 effects and larger P600 effects appearing with increasing proficiency. In visual narratives, recent work has also emphasized the role of proficiency in neural response patterns. We thus explored whether individual differences in proficiency modulate neural responses to visual narrative sequencing in similar ways as in language. We combined ERP data from 12 studies examining semantic and/or grammatical processing of visual narrative sequences. Using linear mixed effects modeling, we demonstrate differential effects of visual language proficiency and “age of acquisition” on N400 and P600 responses. Our results align with those reported in language contexts, providing further evidence for the similarity of linguistic and visual narrative processing, and emphasize the role of both proficiency and age of acquisition in visual narrative comprehension.

**Supplementary Information:**

The online version contains supplementary material available at 10.3758/s13423-023-02334-x.

## Introduction

Recent research has suggested that the comprehension of narrative visual sequences, as found in comics, engages similar cognitive mechanisms to language (Cohn, 2020a). As measured by event-related potentials (ERPs), these manifest as brainwaves with a negative deflection (N400, LAN) and subsequent positivities (P600). While these components are hypothesized as having distinct functions (Baggio, 2018; Brouwer et al., 2017; Kuperberg, 2021), findings also suggest that they differentially arise across participants for any given stimuli (Tanner, 2019; Tanner et al., 2014; Tanner & van Hell, 2014). Indeed, in contexts like language learning, proficiency modulates brain responses, with greater negativities to less proficiency and greater positivities to more proficiency (Steinhauer et al., 2009). Recent work has also shown that proficiency modulates the comprehension of visual narratives based on age and exposure (Cohn, 2020a). Thus, given the similarities in neural responses to language and visual narrative sequencing, here we ask: does proficiency modulate the neural response to visual narrative sequencing?

### Common event-related potential (ERP) metrics of semantic and grammatical processing across modalities

Research using ERPs has revealed several consistent neural responses that appear to both language and visual narratives. The first neural response identified in language processing was the N400, a negative deflection peaking at 400 ms that appeared to violations of meaning in sentence processing (Kutas & Hillyard, 1980). The N400 has thus been taken as an index for the default response relating to semantic access, retrieval, and/or integration (Baggio, 2018; Kutas & Federmeier, 2011; Nieuwland et al., 2020). As such, the N400 is not just sensitive to semantic violations, but also to the graded expectancy of an upcoming word in a sentence (Kutas & Federmeier, 2011). However, N400s do not just appear to manipulations of semantic processing in language, but to all meaningful stimuli. In studies of visual narrative processing, N400s have appeared to incongruous compared to congruous images in a sequence (Coderre et al., 2018; West & Holcomb, 2002) or violations of the semantic congruity of a global sequence (Cohn et al., 2012). N400s to visual narratives are also sensitive to the graded expectancy of an upcoming event, even when they are congruous (Coderre et al., 2020).

In language, functionally distinct neural responses appear in response to manipulations of grammatical structure compared to those of semantics (Morgan et al., 2020). First, a left anterior negativity (LAN), occurring at approximately 100–500 ms, has been observed in response to processing constituent structure (Neville et al., 1991), distance dependencies (Kluender & Kutas, 1993), and violated structural expectancies (Lau et al., 2006; Yano, 2018). A later positivity appearing at approximately 600 ms, the P600, has been most commonly associated with syntactic processing. The P600 first appeared in violations of syntax, such as with garden-path sentences (Osterhout & Holcomb, 1992), violated constituent structures (Hagoort et al., 1993), and morphosyntactic violations (Osterhout & Nicol, 1999). Nevertheless, further experimentation revealed that P600s also appear in semantic contexts, such as violations of argument structure where the grammar remained well formed (Kim & Osterhout, 2005; Kuperberg et al., 2003). Such findings suggested that the P600 reflected a more general process involving integration, updating, or revision of a context given incoming information (Baggio, 2018; Brouwer et al., 2017; Kuperberg, 2021).

Like the syntactic structure of language, visual narratives have been argued to use a “narrative grammar” that uses similar architectural principles to syntax (e.g., categorical roles, recursive constituent structures) in order to package semantic information at a discourse level (Cohn, 2020a). Manipulation of this narrative grammar using linguistic paradigms has comparably evoked LANs and P600s. For example, anterior negativities, often with a left lateralization, have appeared to violations of narrative patterns (Cohn & Kutas, 2017), and to the disruption of constituent structures (Cohn et al., 2014). This anterior negativity also appears insensitive to semantic violations (Cohn & Kutas, 2017), just as the N400 appears to be insensitive to this narrative structure (Cohn et al., 2012).

As with sentence processing, P600s have appeared to manipulations of both meaning and grammar in visual narratives. Larger P600s have appeared in semantic contexts with both congruous and incongruous situational changes between panels (Cohn & Kutas, 2015, 2017), and to processing of sequences with constrained viewpoints on the primary actions (Cohn & Foulsham, 2020). Meanwhile, manipulations of narrative structure have evoked P600s in contexts implying the need for a structural revision of the constituent structure (Cohn et al., 2014; Cohn & Kutas, 2017), and when panels violate the expected narrative categories (Cohn & Kutas, 2015). Such findings reinforce that P600s appear in circumstances requiring backward-looking processes that extend across both grammar and semantics.

### Effects of proficiency and age of acquisition (AoA)

In the language learning literature, it has long been questioned whether second language (L2) learners can ever achieve native-like processing, particularly with regards to morphosyntax. Two main variables have been proposed to drive processing mechanisms: proficiency and age of acquisition (AoA).

#### Proficiency

ERP studies of L2 processing have found that proficiency plays a significant role in modulating N400 and P600 responses. In general, these studies have documented an overall pattern of greater N400 responses with lower proficiency, but greater biphasic LAN-P600^1^ responses with higher proficiency (Caffarra et al., 2015; Steinhauer et al., 2009). Other studies have suggested that alternative variables related to proficiency, like exposure to the L2, modulate this relationship more than proficiency (Fromont et al., 2020a*, *b). Nevertheless, the general pattern of greater N400 effects for lower proficiency in a language and greater P600 effects with higher proficiency seems to be consistent across studies (Caffarra et al., 2015; Steinhauer et al., 2009; Tanner et al., 2013).

This effect is also seen for both semantic and syntactic processing in the native language: lower proficiency is associated with more negativity-dominant ERP responses, while higher proficiency is associated with greater P600 responses (Kasparian et al., 2017; Newman et al., 2012; Pakulak & Neville, 2010; Steinhauer et al., 2009). Thus, the trade-off between negativity-dominant and positivity-dominant responses is not restricted to language learning but can also be observed in native language contexts.

This trade-off of negativity-dominant versus positivity-dominant neural responses is also apparent in findings of a negative correlation between N400 and P600 effect magnitudes (Fromont et al., 2020a, b; Kim et al., 2018; O’Rourke & Colflesh, 2015; Pélissier, 2020; Tanner, 2019; Tanner et al., 2013, 2014; Tanner & van Hell, 2014), creating an N400-P600 “response dominance continuum” (Tanner et al., 2014, p. 287). Individual differences along the continuum have been proposed to reflect variability in sentence processing mechanisms, with individuals who are N400-dominant relying more on memory-based heuristics and those who are P600-dominant relying more on procedural or combinatorial processing (Fromont, Royle, et al., 2020; Tanner et al., 2014). This is in line with some models of language learning (Ullman, 2001), which propose that with increasing proficiency comes a shift from declarative to procedural memory processes.

While acceptance of variation in language proficiency is widespread, the comprehension of visual narratives has been broadly assumed as universal (McCloud, 1993). However, integration of research across disciplines suggests that proficiency in sequential image understanding also requires experience with visual narratives, and may be modulated by the specific types of visual narratives a person is exposed to (see Cohn, 2020a, for a review). Indeed, individuals with little experience with visual narratives have difficulty construing sequential images as a sequence. This ability appears to begin between the ages of 4 and 6 years, when children begin to connect information across images as representing the same referential entities in different states.

Variation in visual narrative proficiency has recently become measurable using the Visual Language Fluency Index (VLFI, pronounced “vil-fee”) questionnaire, which asks participants about their frequency of reading and expertise in visual narratives (comic books and strips, graphic novels, Japanese manga), along with the age that they began reading and drawing comics. These values are then incorporated into the VLFI score, which has been shown to correlate with both behavioral and neurocognitive measures of visual narrative comprehension (see Cohn, 2020a for a review). While this research has suggested neurocognitive differences between comprehenders based on visual narrative proficiency, it has all been done on a study-by-study basis.

#### AoA

AoA effects have been explored not just in the L2 literature but also in L1 learning in the context of sign language. Sign language offers a unique case for investigating the effects of AoA in the absence of other linguistic input because deaf children are often not exposed to either spoken language or sign language until they enter school. However, evidence in both the L2 and sign language fields is mixed regarding the specific influence of AoA.

Some studies do report influences of AoA on the N400 and/or P600 response. For instance, in the L2 domain, Pakulak and Neville (2011) attempted to isolate the effects of AoA by comparing native English speakers with those who learned English later in life, but who were matched on English proficiency. In response to phrase structure violations, the native group showed a biphasic pattern of an early anterior negativity followed by a P600. In contrast, the L2 group showed only a P600 response. Similarly, Nichols and Joanisse (2019) found that later AoA was associated with smaller LANs in response to gender violations. These studies suggest that later AoA is associated with smaller negativity effects.

Other studies have found the opposite pattern of effects. Meulman et al. (2015) found that when high-proficiency bilinguals listened to sentences in their L2 containing gender agreement errors, earlier learners showed a P600 effect while later learners showed a posterior negativity. Similarly, Tanner et al. (2014) found that earlier AoA was associated with more positivity dominance, i.e., a more P600-like profile of processing. In a group of proficient users of Austrian Sign Language with varying AoAs, Malaia et al. (2020) observed a negative correlation between AoA and N400 amplitude for marked word order; that is, later learners showed more negative N400 amplitudes. These studies suggest that later AoA is associated with larger negativity effects while earlier AoA is associated with larger positivity effects.

Finally, other studies have found no effects of AoA on N400 and/or P600 effects. For instance, in the L2 domain, Fromont et al. (2020a, b) did not report any effects of AoA on syntactic category errors in L2 learners. Rather, proficiency was the much larger influencer of N400 and P600 effects. In the sign language domain, Neville et al. (1997) found no effects of AoA on N400 amplitude in response to open-class linguistic elements or semantic anomalies between native English speakers and native ASL signers compared to late deaf learners of English and late hearing learners of ASL.

The specific influence of AoA on the N400 and P600 effects is thus mixed. However, studies that examine both proficiency and AoA generally agree that these two variables are related yet independent (e.g., Newman et al., 2012), and emphasize the importance of measuring both when investigating individual differences.

In visual narratives, the age at which an individual begins reading comics can be conceptualized as an estimate for their “age of acquisition” in much the same way as language. The VLFI assesses not just participants’ overall proficiency based on the frequency that they read comics as a child and as an adult, but also the age at which they started reading comics. Here, we use this metric (hereafter “ASCR,” for “age started comic reading”) as a proxy for AoA to examine the influences of early versus late comic reading on N400 and P600 responses to visual narratives.

### The current study

In sum, evidence from psycholinguistic studies in both the L1 and L2 have demonstrated a consistent trade-off between N400 and P600 responses that is modulated by language proficiency. Because visual narrative processing relies on similar neurocognitive mechanisms to language, and is similarly modulated by proficiency, we here ask two primary questions: (1) Do the neural responses to visual narratives exhibit a trade-off between the N400 and P600 ERP components, as found in language research? (2) If so, might these neural response patterns be modulated by proficiency and AoA in visual narratives?

To answer these questions, we performed a comprehensive analysis of 12 studies examining various aspects of visual narrative processing using ERPs that also included a measure of comic reading proficiency. For each study, we quantified the N400 and P600 effect magnitudes for each participant across a variety of different types of contrasts tapping semantic, grammatical, and both semantic and grammatical processing. To briefly foreshadow the results, our linear mixed effects models showed that visual language proficiency modulates N400 ERP magnitude in similar ways to in the language literature. Furthermore, we observed consistent interactions of proficiency with AoA, which highlights the importance of considering both factors when assessing “fluency.” Overall, these results provide further evidence for the similarity of linguistic and visual narrative processing and for the role of proficiency in visual narrative comprehension.

## Methods

### Studies

A full list of all studies and contrasts included in the analyses is provided in Table 1. More detailed information on the background and general methods for each study can be found in Online Supplementary Material (OSM) 1, and in the associated published reports (when applicable). Only studies that used EEG to investigate visual narrative processing, and also included a measure of comic proficiency via the VLFI, were included. In total, we here analyze data from 12 different studies testing 286 subjects (mean age = 22.4 years, SD = 6.7; 132 males, 154 females). Across all studies there were 23 different contrasts included. Contrasts were categorized into “contrast types” according to whether they examined semantic processing, grammatical processing, or both semantic and grammatical processing.

**Table 1:** List of studies included in the analyses, with descriptions of the contrasts and “contrast type” category

Study	N	N400 time window	P600 time window	Contrast(s)		Contrast type	Reported effect
				“Ungrammatical”	“Grammatical”		
Cohn et al. (2012)	24	400–600 ms	600–900 ms	Scrambled	Normal	Semantic + grammatical	N400
				Structural	Normal	Semantic	N400
				Semantic	Normal	Grammatical	N400
				Scrambled	Semantic	Semantic	N400
				Scrambled	Structural	Grammatical	LAN
Cohn (2012)	32	400–600 ms	600–900 ms	Dual	Normal	Semantic + grammatical	N400
				Narrative	Normal	semantic	N400
				Semantic	Normal	grammatical	N400
				Scrambled	Semantic	grammatical	N400
				Scrambled	Narrative	Semantic	N400
Cohn et al. (2014)	24	300–500 ms	500–700 ms	Within-Second Constituent	Between Constituents	Grammatical	LAN P600
Cohn & Kutas (2015)	36	400–600 ms	600–800 ms	Impoverished (at critical panel)	Explicit (at critical panel)	Grammatical	P600
				Impoverished (at critical panel + 1)	Expected (at critical panel + 1)	Semantic + grammatical	LAN P600
Cohn & Kutas (2017)	28	300–500 ms	500–700 ms	Non-conjunction	Conjunction	grammatical	LAN P600
Coderre et al. (2018)	20	300–500 ms	500–800 ms	Incongruent	Congruent	Semantic	N400
Coderre et al. (2020)	22	300–500 ms	500–800 ms	Anomalous	High cloze	Semantic	N400
Cohn (2021)	24	300–500 ms	500–800 ms	Action star	Explicit	Semantic	N400
				Noise	Action star	Grammatical	P600
				Noise	Explicit	Grammatical	N400
Cohn & Foulsham (2020)	24	300–500 ms	500–700 ms	Zoom zoom	Normal full	Semantic	N400
Coopmans & Cohn (2022)	32	300–500 ms	500–800 ms	Anaphoric distant	Anaphoric proximal	grammatical	N400
Cohn & Foulsham (in preparation)	32	300–500 ms	500–800 ms	Incongruous Zoom	Congruous Full-Scene	Semantic	N400
Pellegrino-Wood et al. (in preparation)	20	400–600 ms	600–900 ms	Scrambled	Normal	Semantic + grammatical	N400
				Structural	Normal	Semantic	N400
				Semantic	Normal	Grammatical	N400
				Scrambled	Semantic	Semantic	N400
				Scrambled	Structural	Grammatical	N400 P600

### N400 and P600 effect calculations

To assess effects of proficiency and AoA on the N400 and P600 ERP responses, we quantified the N400 and P600 “effects” in the following way, as defined in Tanner et al. (2014):

1 $$N400\;effect={N400}_{Gram}- {N400}_{Ungram}$$

2 $$P600\;effect= {P600}_{Ungram}- {P600}_{Gram}$$

In these equations, the “ungrammatical” condition is meant to be the one that elicits the larger (positive or negative) amplitudes compared to the “grammatical” condition. Although we investigate both semantic and grammatical contrast types in the current analyses, we maintain these labels when describing the condition comparisons in Table 1 to facilitate interpretation of our mathematical procedures. (Note that the “N400 effect” is calculated as “grammatical minus ungrammatical,” meaning that larger N400 effects means a more negative amplitude of the “ungrammatical” condition.) For all studies, the average ERP amplitude for the conditions of interest was calculated over the specified time window and over central and parietal sites (electrodes C3, Cz, C4, P3, Pz, and P4).

### Visual Language Fluency Index (VLFI) analysis

Experience and proficiency with visual narratives like comics were assessed across all studies using the VLFI questionnaire (all VLFI materials are available in OSM 2, or downloadable at www.visuallanguagelab.com/vlfi). This survey asks participants to rate their frequency of reading a variety of visual narratives (comic strips, comic books, graphic novels, Japanese manga) on a 1 (never) to 7 (always) scale, both for their “current” reading habits and “while growing up.” They are also asked to rate their comic reading expertise for both time periods (1 = below average, 5 = above average). Frequency of drawing comics and expertise rating for drawing ability are also asked. This information is compiled into a calculation that gives a “VLFI Score”:

3 $$VLFI\;Score= \left(\begin{array}{c}Mean\;Comic\;Reading\;Freq. \\ \times Comic\;Reading\;Expertise\end{array}\right)+\left(\frac{Comic\;Drawing\;Freq. \times Drawing\;Ability}{2}\right)$$

An analysis of almost 2,000 VLFI surveys (Cohn, 2020a) has shown an average VLFI score of 15.16 (SD = 9.6), which is in line with the idealized average falling between 12 and 20, with low being below 12 and high being above 20 (maximum possible = 52). Across all of the studies analyzed here, participants had an average VLFI score of 13.94 (SD = 6.7), with a range of 2–35.25. For the purposes of regression analyses, VLFI scores were centered to have a mean of 0.

The VLFI also asks participants to provide the ages at which they started reading and drawing comics. Across all of the studies analyzed here, participants had a mean age of starting to read comics of 8.23 years (SD = 2.9, with nine subjects reporting “N/A”) and of starting to draw comics at 8.91 years (SD = 3.8, with 96 subjects reporting “N/A”). These ages are consistent with those from the aggregated analysis of VLFI surveys, where average age of starting to read comics was 8.38 (SD = 3.4), and age of starting to draw comics was 9.83 (SD = 4.2). For subsequent analyses, subjects who reported “N/A” for “age started comic reading” (ASCR) were removed; this ensured that analyses of VLFI and ASCR were performed using the same data.

### Statistical analyses

Linear mixed effects modeling was performed with the *lme4* package (version 1.1-27, Bates et al., 2020) in R version 4.2.2 (R Core Team, 2022). *Subject* and *study* were included as random intercepts. The reference level for the *contrast type* variable was set as the “semantic + grammatical” contrast; sum coding was used to estimate effects for this categorical variable. *VLFI* used the centered VLFI scores in all models. In investigating each ERP response (N400 or P600) as an outcome measure, we included the other ERP effect as a covariate. That is, for models with the *N400 effect* as an outcome measure, *P600 effect* was included as a covariate, and vice versa, to account for the influences of these two ERP components on each other.

We also included *N400 time window* and *P600 time window* as covariates (fixed effects), since these differed between studies. Rather than choose a standardized window for each effect across all studies, we chose to calculate the N400 and P600 effects based on the time windows in which each ERP effect was identified in the original study. For instance, if one study showed the strongest N400 effect from 300–400 ms and another from 400–500 ms, then using the same window for both (e.g., 300–500 ms) would diminish the effects of each and misrepresent the N400 magnitude for both studies. The studies in our dataset contained two different N400 time window: 300–500 ms and 400–600 ms; the 300–500 ms level was set as the reference level for modeling purposes. Our dataset also contained four different P600 time windows: 500–700 ms, 500–800 ms, 600–800 ms, and 600–900 ms; the 500–700 ms level was set as the reference level. Note that this does introduce rank deficiency into the modeling for the P600 time windows, since all of the studies with a 600–900 ms P600 window also have a 400–600 ms N400 window (see Table 1), and therefore all of the variability that is potentially captured by the 600–900 ms P600 time window has already been explained by the 400–600 ms N400 window. Because the additional data do not help estimate the model, these data are “dropped” in R, which is why the model results do not include output for the 600–900 ms P600 window. While rank deficiency is not a serious issue for model estimation (the *lmer* package gives a warning message, not an error), we mention this issue to assist with interpreting the model outputs.

All models were estimated using maximum likelihood. *t*-scores greater than 2 were interpreted as significant effects corresponding to an alpha of 0.05 or lower (Meier & Kane, 2013; see also Gelman et al., 2012). The *lmerTest* package (Kuznetsova et al., 2017) was also used to generate *p*-value estimates. Only significant main effects of or interactions with VLFI and/or ASCR were followed up. Interactions between VLFI and ASCR were visualized using the *interact_plot()* function in the *interactions* package in R. Raw data and source code are provided in OSM 3.

## Results

### N400 versus P600 effects

**Fig. 1:**
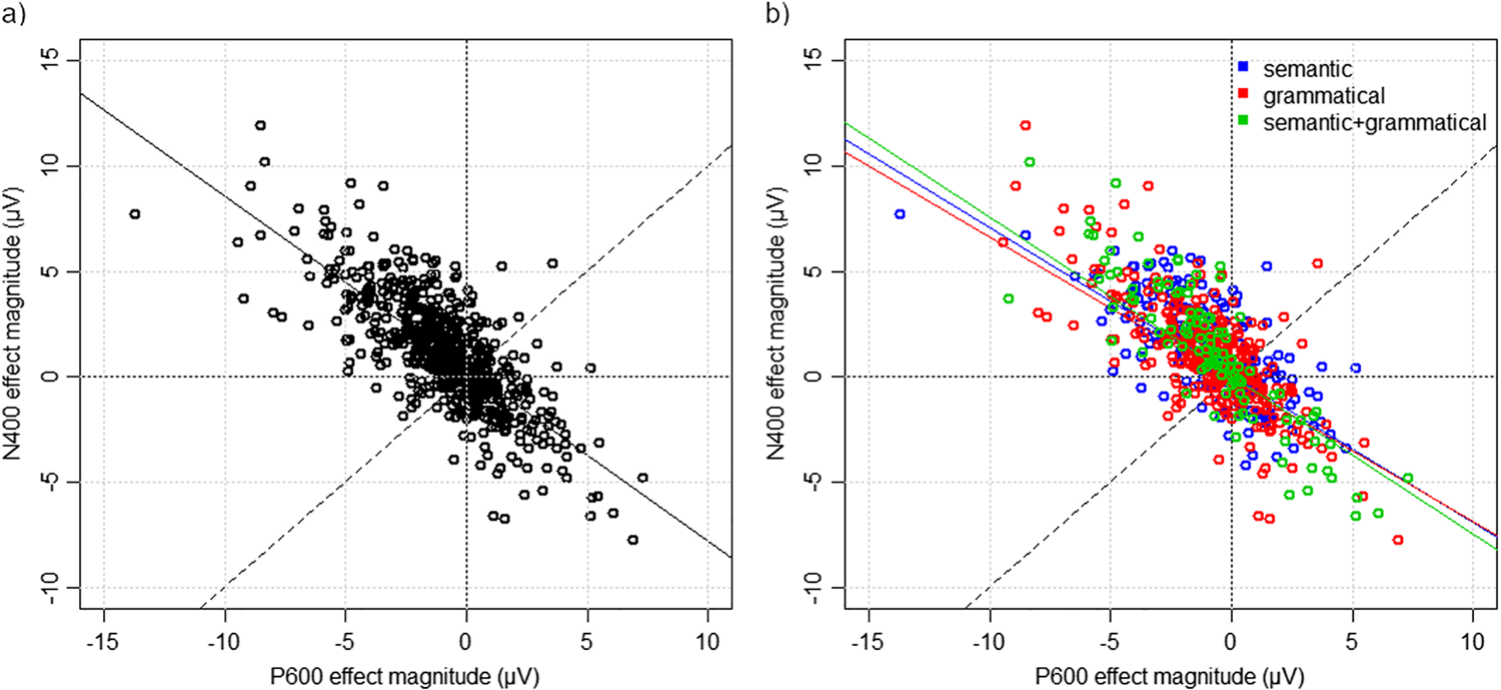
Scatterplots of N400 effect magnitudes against P600 effect magnitudes, for (**a**) all contrasts and (**b**) each contrast type. The dashed line represents equal N400 and P600 magnitudes. Values above the dashed line indicate a negativity-dominance, i.e., participants primarily show an N400 effect to the various contrast types. Values below the dashed line indicate a positivity-dominance, i.e., participants primarily show a P600 effect to the various contrast types. The solid diagonal lines indicate the best-fit line from the correlation analysis for each contrast type. Each dot represents one participant

To first explore the trade-off between N400 and P600 effects among all participants and all studies/contrasts, we plotted the N400 effect magnitude against the P600 effect magnitude. As can be seen in Fig. 1a, over all contrasts there was a significant negative correlation (*r* = -0.76, *p* < 0.001), such that individuals who showed a larger N400 effect tended to show a smaller P600 effect, and vice versa. We also plotted this comparison for each contrast type individually; as seen in Fig. 1b, there were significant negative correlations for the semantic (*r* = -0.69, *p* < 0.001), grammatical (*r* = -0.74, *p* < 0.001), and semantic + grammatical (*r* = -0.87, *p* < 0.001) contrast types. These findings replicate those of Tanner and van Hell (2014) in demonstrating that both semantic and grammatical processing persist along a continuum of negativity and positivity responses which vary across individuals.

### Effects of proficiency and AoA

We next turned to examining the effects of visual language proficiency (VLFI scores) and age of acquisition (ASCR) on the N400 and P600.

#### N400 effects

To determine whether VLFI and ASCR had differential effects on the N400 effect, we first ran a model using *N400 effect* as an outcome variable and including fixed effects of *VLFI score*, *ASCR*, and *contrast type* (semantic, grammatical, or semantic + grammatical). ASCR and VLFI scores were not strongly correlated in our dataset: correlating these two metrics yielded an *r*-value of -0.08. Although this is statistically significant (*p* < 0.05) due to the large number of datapoints, by any metrics of interpretation this *r*-value reflects a negligible correlation. *P600 effect* was included as a covariate. As in all models, N400 and P600 *time windows* were also included as covariates (fixed effects) and *subject* and *study* were included as random intercepts:

$$N400 effect \sim VLFI * ASCR * contrast type + P600 effect + N400 time window + P600 time window +\left(1|subject\right)+ \left(1|study\right)$$

Note that because the reference level for *contrast type* is the ‘semantic + grammatical’ contrast, effects of *contrast type: grammatical* indicate a significant difference between the semantic + grammatical and grammatical contrasts, while effects of *contrast type: semantic* indicate a difference between semantic and semantic + grammatical contrasts.

**Fig. 2:**
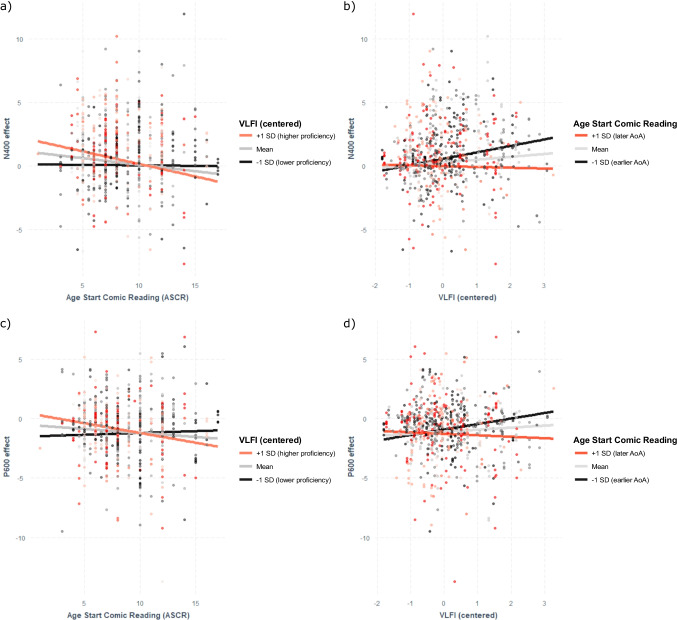
Interaction plots for the models of N400 effects (top row) and P600 effects (bottom rows), broken up by high/low VLFI (left panels), and early/late ASCR (right panels)

The full results are presented in Table 2. We observed a significant effect of VLFI but not ASCR, although there was an interaction between VLFI and ASCR. As can be seen in Fig. 2 (panels a and b), participants with low proficiency showed little modulation of the N400 effect by AoA. In contrast, participants with high proficiency showed a large influence of AoA, with earlier AoA associated with larger N400 effects and later AoA associated with smaller N400 effects. Furthermore, among late learners, lower proficiency was associated with larger N400 effects.

**Table 2:** Results of the linear mixed effects model with *N400 effect* as an outcome variable and *VLFI*, *ASCR*, and *contrast type* as fixed effects. The reference level for *contrast type* is the semantic + grammatical contrast. *P600 effect* and N400 and P600 *time windows* are included as covariates (fixed effects) and *subject* and *study* are included as random intercepts. Trends and significant effects are indicated with asterisks (§ = *p* < 0.10; * = *p* < 0.05; ** = *p* < 0.01; *** = *p* < 0.001)

Model formula:					
N400 effect ~ P600 effect + N400timewindow + P600timewindow + VLFI * ASCR * contrast type + (1|subject) + (1|study)					
AIC: 2515.8					
Random effects		Variance	SD		
Subject	Intercept	0.51	0.72		
Study	Intercept	0.05	0.22		
Residual		1.81	1.35		
Fixed effects	Estimate	SE	Df	*t*-value	*p*-value
(Intercept)	-0.24	0.32	61	-0.77	0.44
N400 time window: 400–600	1.76	0.28	11	4.15	<0.01 **
P600 time window: 500–800	1.03	0.28	16	3.63	<0.01 **
P600 time window: 600–800	-1.00	0.35	7	-2.85	0.03 *
P600 effect	-0.81	0.03	662	-31.55	<0.001 ***
VLFI	0.67	0.24	259	2.79	<0.01 **
ASCR	-0.03	0.03	266	-1.14	0.26
Contrast type: grammatical	0.70	0.29	506	2.40	0.02 *
Contrast type: semantic	-0.76	0.22	492	-3.41	<0.001 ***
VLFI*ASCR	-0.06	0.03	260	-2.17	0.03 *
VLFI*contrast type: grammatical	0.36	0.30	503	1.23	0.22
VLFI*contrast type: semantic	-0.24	0.23	502	-1.06	0.29
ASCR*contrast type: grammatical	-0.07	0.03	517	-2.08	0.04 *
ASCR*contrast type: semantic	0.07	0.03	498	2.97	<0.01 **
VLFI*ASCR*contrast type: grammatical	-0.04	0.03	504	-1.10	0.27
VLFI*ASCR*contrast type: semantic	0.03	0.03	514	0.97	0.33

**Fig. 3:**
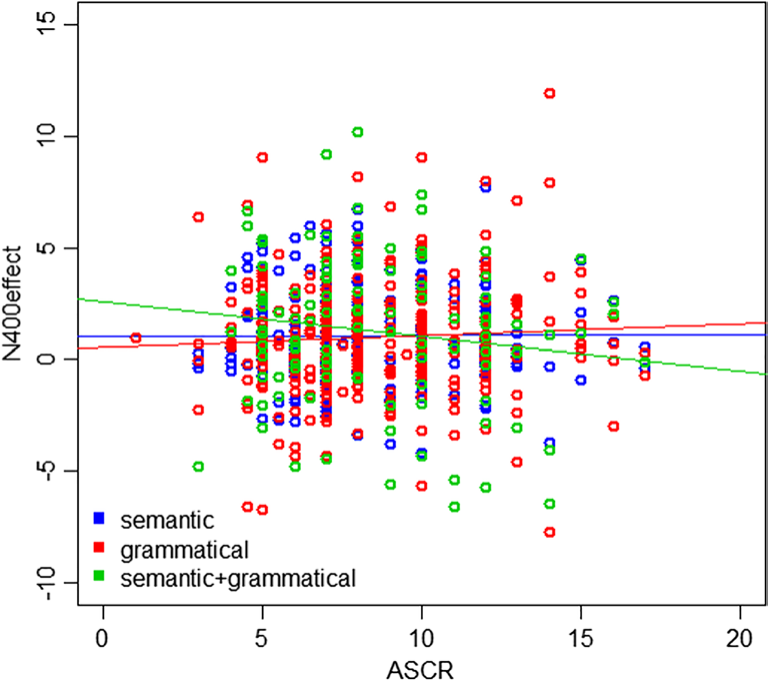
Scatterplot of N400 effect against ASCR for each of the three contrasts. Each dot represents one participant

Interactions of ASCR with contrast type were explored by plotting ASCR against N400 effects for each contrast type (Fig. 3). The semantic only (*r* = 0.002, *p* = 0.98) and grammatical only (*r* = 0.06, *p* = 0.25) contrasts showed negligible positive correlations, while the semantic + grammatical contrast showed a negligible negative correlation (*r* = -0.08, *p* = 0.58). Although no correlations were significant, Fig. 3 suggests that the presence of both semantics and grammar elicits slightly different patterns compared to the influence of either factor on its own.

#### P600 effects

To determine whether VLFI and ASCR had differential effects on the P600 effect, we next ran a model using *P600 effect* as an outcome variable and including fixed effects of *VLFI score*, *ASCR*, and *contrast type* (semantic, grammatical, or semantic + grammatical). *N400 effect* was included as a covariate. As in all models, N400 and P600 *time windows* were also included as covariates (fixed effects) and *subject* and *study* were included as random intercepts:

$$N600 effect \sim VLFI * ASCR * contrast type + P400 effect + N400 time window + P600 time window +\left(1|subject\right)+ \left(1|study\right)$$

The full results are shown in Table 3. Here, we observed an effect of VLFI but not ASCR, and an interaction of VLFI and ASCR. As can be seen in Fig. 2 (panels c and d), participants with low proficiency did not show much modulation of the P600 effect by AoA. However, participants with high proficiency showed a large influence of AoA, with early AoA associated with larger P600 effects. Among late learners, higher proficiency was associated with smaller P600 effects.

**Table 3:** Results of the linear mixed effects model with *P600 effect* as an outcome variable and *VLFI*, *ASCR*, and *contrast type* as fixed effects. The reference level for *contrast type* is the semantic + grammatical contrast. *N400 effect* and N400 and P600 *time windows* are included as covariates (fixed effects) and *subject* and *study* are included as random intercepts. Trends and significant effects are indicated with asterisks (§ = *p* < 0.10; * = *p* < 0.05; ** = *p* < 0.01; *** = *p* < 0.001)

Model formula:					
P600 effect ~ N400 effect + N400timewindow + P600timewindow + VLFI * ASCR * contrast type + (1|subject) + (1|study)					
AIC: 2452.7					
Random effects		Variance	SD		
Subject	Intercept	0.49	0.70		
Study	Intercept	0.03	0.17		
Residual		1.64	1.28		
Fixed effects	Estimate	SE	Df	*t*-value	*p*-value
(Intercept)	-0.19	0.29	76	-0.63	0.53
N400 time window: 400–600	0.51	0.25	13	2.01	0.07 §
P600 time window: 500–800	0.77	0.26	18	3.03	0.01 **
P600 time window: 600–800	-0.56	0.31	7	-1.82	0.11
N400 effect	-0.74	0.02	657	-31.60	<0.001 ***
VLFI	0.61	0.23	259	2.67	0.01 **
ASCR	-0.03	0.03	264	-1.29	0.20
Contrast type: grammatical	0.37	0.28	500	1.32	0.19
Contrast type: semantic	-0.49	0.21	488	-2.29	0.02 *
VLFI*ASCR	-0.06	0.03	261	-2.28	0.02 *
VLFI*contrast type: grammatical	0.38	0.28	495	1.34	0.18
VLFI*contrast type: semantic	-0.28	0.22	495	-1.28	0.21
ASCR*contrast type: grammatical	-0.03	0.03	511	-0.92	0.36
ASCR*contrast type: semantic	0.04	0.02	491	1.81	0.07 §
VLFI*ASCR*contrast type: grammatical	-0.04	0.03	495	-1.19	0.24
VLFI*ASCR*contrast type: semantic	0.03	0.02	506	1.12	0.26

**Fig. 4:**
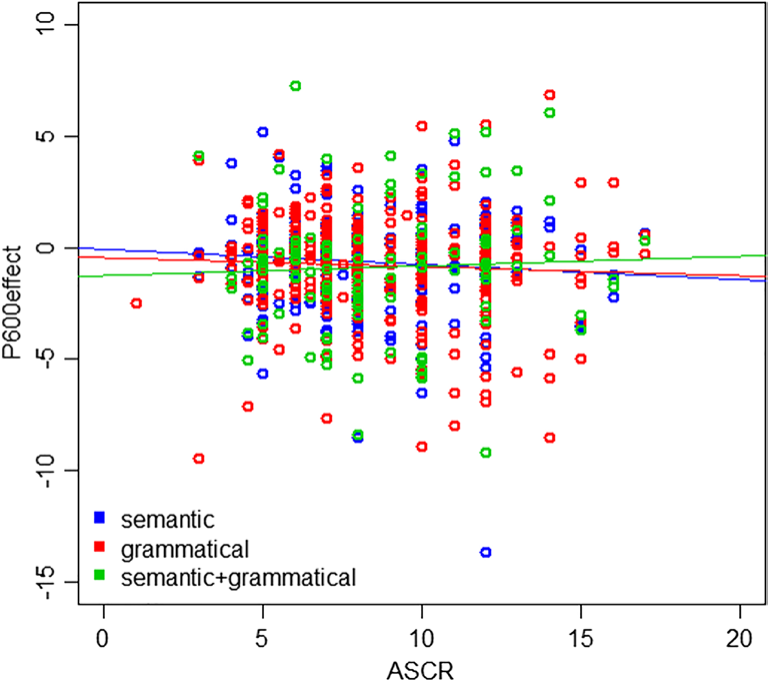
Scatterplot of P600 effect against ASCR for each of the three contrasts. Each dot represents one participant

The trend of an interaction of ASCR with contrast type was explored by plotting ASCR against P600 effects for each contrast type (Fig. 4). The semantic only (*r* = -0.09, *p* = 0.17) and grammatical only (*r* = -0.05, *p* = 0.35) contrasts showed negligible negative correlations, while the semantic + grammatical contrast showed a negligible positive correlation (*r* = 0.05, *p* = 0.64). Although no correlations were significant, this visualization suggests that the presence of both semantics and grammar elicits slightly different patterns compared to the influence of either factor on its own.

## Discussion

Psycholinguistic studies have suggested that increasing proficiency (in both the native language and a second language) is associated with a general shift from more negative, N400-like neural responses to more positive, P600-like responses (Fromont et al., 2020a, b; Kim et al., 2018; O’Rourke & Colflesh, 2005; Pélissier, 2020; Tanner, 2019; Tanner et al., 2013, 2014; Tanner & van Hell, 2014). Proficiency also modulates neural indices of visual sequence processing (Cohn, 2020a); however, whether comic reading proficiency causes the same negativity-to-positivity pattern had never been investigated. This study integrated the results from 12 ERP studies on visual narratives to examine whether proficiency with comic reading modulates neural response patterns to semantic and grammatical processing.

### The response dominance continuum

The current study replicated previous findings of a negative correlation between N400 and P600 effects, such that participants who showed larger N400s tended to show smaller P600s, and vice versa. In fact, this relationship was one of the strongest shown thus far in any prior studies: we observed a marked negative relationship (*r* = -0.76), whereas other studies have shown correlation coefficients in the range of approximately -0.4 to -0.7 (Fromont et al., 2020a, b; Kim et al., 2018; Pélissier, 2020; Tanner, 2019; Tanner et al., 2013, 2014; Tanner & van Hell, 2014). To our knowledge, the only other study reporting a stronger correlation was O’Rourke and Colflesh (2005), who reported a correlation coefficient of -0.9, in a task assessing comprehension of garden-path sentences in 60 participants. The robust effect seen in our data may be a result of the larger number of subjects included in this aggregate of multiple ERP studies. Nevertheless, these results confirm previous proposals that individuals vary along an N400-to-P600 “response dominance continuum” in processing strategies, and work even showing that N400 and P600 effects are coupled in the individual responses to trials (Aurnhammer et al., 2023).

Most importantly, we demonstrate for the first time that this relationship in processing mechanisms is not restricted to verbal language but is also present for visual narrative comprehension. Prior studies of visual narratives (and other meaning-making domains) have claimed to elicit ERP components as the same as those first discovered in language (i.e., N400, LAN, P600). These results provide evidence that verbal and visual languages indeed rely on similar processing mechanisms, or at least that such mechanisms demonstrate similar tendencies in their dominance continuum.

#### Effects of proficiency and AoA

We also explored how metrics of proficiency and AoA modulated the magnitude of the N400 and P600 ERP responses in our data. VLFI scores aggregate self-reported frequency and expertise of comic reading and drawing, and so can be conceptualized as a metric of “proficiency” with the visual languages used in comics, similar to proficiency in spoken language. On the other hand, the age that an individual begins to read comics (ASCR) can be conceptualized as a metric of AoA, again similar to that of language. Although proficiency and AoA are often correlated in verbal language, they are dissociable (e.g., an individual may have begun speaking their L2 later in life but reach very high proficiency levels), and presumably the same is true for visual languages.

VLFI and ASCR interacted in their modulations on both the N400 and P600 effects. The overall patterns of results were similar for both the N400 and P600 components: AoA had the largest influence for individuals with higher proficiency. This makes sense, as those with low proficiency may not have acquired the basic patterns of comic reading to elicit large N400s or P600s. It is only when proficiency reaches a certain level of proficiency that the influences of AoA become apparent.

Among later learners, lower proficiency was associated with larger N400 effects, which is in line with previous research (Caffarra et al., 2015; Steinhauer et al., 2009; Tanner et al., 2013). However, this pattern becomes more nuanced when looking at the interaction between proficiency and AoA. For later learners, proficiency made no modulation of the N400, while for early learners, *higher* proficiency was associated with larger N400 effects. This discrepancy points to the importance of examining both proficiency *and* AoA when investigating individual differences in narrative comprehension.

A similar pattern occurred for the P600: proficiency did not modulate the P600 among late learners, but among early learners, greater proficiency was associated with larger P600 effects, in addition to larger N400 effects. This is contradictory to previous research, which has reported a trade-off such that lower proficiency is associated with larger N400s and smaller P600s (Caffarra et al., 2015; Steinhauer et al., 2009; Tanner et al., 2013).

In general, the N400 has been associated with more forward-looking processing, while the P600 has been associated with backward-looking processes of reanalysis or updating (Cohn, 2020a; Kotchoubey, 2006). Among participants with higher proficiency, earlier AoA was associated with larger N400 and P600 effects, which could reflect the greater sensitivity to the semantic and grammatical manipulations in the stimuli. Here, violations to visual sequences incur overall more effortful processing when comic reading is familiar (having started early in life and with a high proficiency), caused by the acquisition of top-down expectancies, which in turn are violated in these experimental paradigms. In contrast, lower expertise participants (late learning and/or low proficiency) showed relatively lesser N400 and P600 effects, perhaps suggesting a more uniform bottom-up processing strategy overall.

This is line with the findings of Meulman et al. (2015), who report a P600 effect for earlier L2 learners but a negativity for later learners. However, the L2 literature is mixed with regards to the effect of AoA on P600 effects, with some studies finding smaller negativity effects for later AoA. It is thus difficult to conclude whether our AoA effects fit in with a general pattern of AoA effects in the literature, and it is possible that the overall similar patterns between the effects in our data suggest a different response than a true biphasic N400/P600 in response to visual narratives (see next section). Nevertheless, the fact that distinct patterns were found for early versus late comic readers, and that these patterns also interacted with proficiency, again point to the importance of measuring and considering both variables.

#### Similar patterns of effects between N400 and P600 components

One interesting observation from our data is that similar effects occurred for both the N400 and P600 components: the highest expertise was associated with both larger N400 and larger P600 effects. This is contradictory to previous research, which has generally identified a trade-off such that larger N400s are associated with smaller P600s, and vice versa. We did observe this same continuum, as evidenced by a strong correlation between N400 and P600 responses; however, a closer observation of Fig. 1 suggests that the majority of individuals who show large positive N400 effects show *negative* P600 effects. Because of the way the P600 effect is calculated here, a negative P600 effect is a greater negativity to the violation or more ungrammatical condition: in other words, an N400-like negativity. Thus, one possibility is that these negative P600 effects are actually a sustained negativity that extends into the P600 window. Sustained negativities appear in verbal narratives in contexts of co-reference resolution like anaphoric relations, or in inferencing, possibly tied to sustained working memory processes (Cohn, 2021; Coopmans & Cohn, 2022).

Whereas language stimuli commonly elicit a biphasic N400/P600 complex, visual narratives sometimes elicit sustained negativities and no later positivities (Coderre et al., 2018; Cohn, 2012; Cohn et al., 2012; Coopmans & Cohn, 2022). Our method of calculating N400 and P600 effects was taken from Tanner et al. (2014), who explore the response-dominance continuum; however, examination of their plot of N400 versus P600 magnitudes (i.e., their analogous figure to our Fig. 1; Fig. 2 in their manuscript) identifies the majority of people in the top right quadrant, showing positive N400 effects and P600 effects, thus suggesting this biphasic N400–P600 trade-off that is more traditional in language studies. In contrast, the large distribution of participants in our study who fall in the top left quadrant (positive N400s and negative P600s) or bottom right quadrant (positive P600s but negative N400s) suggests a more sustained effect that is being captured by both time windows. The idea of the response-dominance continuum still holds, since individuals tend to fall on either the “more negative” or “more positive” side of the graph; however, rather than a trade-off between N400 and P600 components specifically, this negativity or positivity seems to extend throughout the duration of narrative processing.

One possibility is that the biphasic N400/P600 response commonly seen for verbal stimuli occurs because words are processed relatively quickly. In contrast, visual images generally have more information to process, which could lead to more prolonged effects like sustained negativities. Indeed, reduced visual information in an image, such as “action stars” in a visual narrative sequence (which are panels with only a star-shaped flash that push a reader to infer the undepicted events), renders a different pattern of ERP effects. In a prior ERP study, action stars showed a more biphasic N400/P600 response compared to traditional comic panels, perhaps because of their relative lack of information processing demands (Cohn, 2021). Whether a sustained negativity is observed may also have to do with the specific experimental manipulation. If a specific manipulation elicits especially large positivities, this may be enough to break through the sustained negativities to create a P600 effect. Although speculative, these interpretations are in line with theoretical models that propose the N400 and P600 are indicative of the same overall process (e.g., Aurnhammer et al., 2023; Baggio, 2018). Taken together, the results of the current study suggest that N400 and P600 ERP responses to visual narratives do seem to be modulated by proficiency and AoA, much like in verbal narratives, but that there are also modality differences in the specific pattern of ERP effects elicited by different contrasts.

#### Effects of contrast type

The 12 ERP studies included in these analyses were originally designed to test various aspects of semantic and grammatical processing in visual narrative sequences. Therefore, in all models, we included “contrast type” as a factor depending on whether the original contrast(s) of interest isolated semantic processing, grammatical processing, or both. These contrasts had originally elicited a range of ERPs including N400s, LANs, and P600s (Table 1). Interestingly, across all analyses, the combined semantic + grammatical contrast appeared to elicit different patterns than the semantic or grammatical alone.

When plotting N400 effects against ASCR scores, the combined contrast showed the strongest relationship of the three different contrast types. When plotting P600 effects against ASCR scores, the semantic + grammatical contrast showed an opposite effect to the other two contrasts individually. The semantic + grammatical contrast also showed the strongest relationship between raw N400 and P600 values, with a correlation coefficient of -0.87 (compared to -0.69 and -0.74 for the semantic and grammatical contrasts, respectively). Thus, there seems to be something different occurring when semantic and grammatical processing are combined, compared to contrasts that isolate one of these functions. This suggests that examining individual differences in proficiency could be informative as to the structure of the processing system, with separable aspects of processing having a different response profile to when both components are concurrently manipulated (Baggio, 2018; Cohn, 2020b).

To begin with, we note that in most cases, the semantic-only and grammatical-only contrasts appeared to be processed very similarly. If an individual uses a particular processing mechanism (e.g., more N400-like vs. more P600-like) to deal with both semantic and grammatical violations when encountered alone, they might also use that same processing mechanism to deal with combined violations. One possibility for the opposite pattern seen in the combined semantic + grammatical conditions is that there could be an additive effect occurring, such that processing two types of violations at once recruits mechanisms for processing each individually in a combinatorial way (Osterhout & Nicol, 1999). This could result in different magnitudes of effects for the combined conditions compared to the semantic or grammatical alone. Another possibility (not necessarily contradictory to the first) is that there is a compensatory effect for the combined violations because of an over-taxing of the system. If a negativity-dominant processing mechanism is recruited to deal with semantic and grammatical violations alone, then encountering both at the same time may overload that processing stream, such that the alternative stream is recruited and the individual shows a more positivity-dominant effect. Although these interpretations are speculative, it is clear from the data that different neural mechanisms are recruited for processing semantic and grammatical contrasts together compared to each individually.

We note that one advantage to the current study is that we include multiple different types of contrasts. While some studies exploring the relationship between N400 and P600 effects have included both semantic and grammatical processing (Fromont et al., 2020a, b; Mehravari et al., 2017), there have been no such studies that compare ERP responses between contrast types, nor investigate how proficiency or AoA differentially affect these responses in different contrasts. Our meta-analytic approach in combining multiple studies testing different aspects of processing provides us with a wider viewpoint and offers intriguing insight into the recruitment, and trade-offs, of semantic and grammatical processing when presented alone versus in combination.

## Conclusions

Altogether, our results suggest that proficiency differences modulate individual neural responses in visual narrative processing in similar ways to language. This offers further evidence that verbal and visual languages are processed using similar neurocognitive mechanisms. It also highlights the importance of considering proficiency more carefully, in both linguistic and visual modalities. For instance, many studies in the language learning literature use a group of native speakers as a control group, whereas there is evidence that even within monolinguals there are individual differences in language proficiency. Investigating individual differences in proficiency may be useful in these situations to fully describe the influence of language experience on neural responses.

This is equally important to consider for visual narratives, which are often used in psychological experimentation as stimuli under the assumption of their transparency (Coderre, 2020; Cohn, 2020a). Although understanding picture sequences is often thought to be innate and universal, our results add further support to a mounting body of evidence showing that visual narrative comprehension is also subject to effects of proficiency and AoA, just as in language. It is thus crucial to consider both variables when using visual narratives for experimental or clinical testing purposes.

Finally, these results offer important evidence of individual differences in proficiency and AoA modulating neural responses to image comprehension. Given the parallels we have drawn between visual and verbal language, these findings are relevant to language processing in any modality. Similar ERP components also appear outside of language, such as for visual event cognition (Amoruso et al., 2013; Sitnikova et al., 2008) and scene perception (Võ, 2021) or musical expertise (Zhang et al., 2016), making these measures of individual differences relevant for studies in those areas as well. Highlighting the continuum of responses suggests that neurocognition of these components is not uniform or universal, but rather involved in dynamic processing mechanisms across, and likely within, individuals. Such variability across neural functions should thus be accountable by neurocognitive models of these domains. By treating individual differences as a source of meaningful variation, rather than a source of error, we can make significant progress in understanding the dynamic processing pathways that underlie verbal and visual language processing.

### Supplementary Information

Below is the link to the electronic supplementary material.

Supplementary file1 (DOC 50 KB)

Supplementary file2 (XLSX 1005 KB)

## Data Availability

All data analyzed for this study are included in Online Supplementary Material 3.

## Footnotes

1 Although see Tanner and van Hell (2014), who argue that the LAN is an artifact of the grand averaging that is typical of ERP analyses, and instead reflects the individual variability in N400-P600 responses.

## Code availability

All source code for the analyses performed in this study are included in Online Supplementary Material 3.
