# Supplementary material for: Individual differences in the neural dynamics of visual narrative comprehension: The effects of proficiency and age of acquisition
Source: Psychon Bull Rev. 2023 Aug 14;31(1):89–103. doi: 10.3758/s13423-023-02334-x (PMC10866750; doi:10.3758/s13423-023-02334-x)
Supplement: Supplementary file 1 — Supplementary file1 (DOC 50 KB) [file 13423_2023_2334_MOESM1_ESM.doc]

1. Using the following scale, on average, how often per week do/did you…? (place a whole number in the square)

Never -----------------------------------Sometimes-----------------------------------Always

1 2 3 4 5 6 7

|  | **Currently**  **(mark 1 – 7)** | **While growing up**  **(mark 1 – 7)** | **Which is your favorite? (mark with “X”)** |
| --- | --- | --- | --- |
| …read text-only books for enjoyment |  |  | DO NOT MARK HERE |
| …watch movies |  |  | DO NOT MARK HERE |
| …watch cartoons/anime |  |  | DO NOT MARK HERE |
| …read comic books |  |  |  |
| …read comic strips |  |  |  |
| …read graphic novels |  |  |  |
| …read Japanese comics (manga) |  |  |  |
| …draw comics |  |  |  |

2. How would you rate your expertise with reading comics (of any sort)? (Mark “X” once in each row)

|  | **Above average (5)** | **Slightly above average (4)** | **Average (3)** | **Slightly below average (2)** | **Below average (1)** |
| --- | --- | --- | --- | --- | --- |
| Currently |  |  |  |  |  |
| While growing up |  |  |  |  |  |

3. How would you rate your drawing ability? (Mark “X” once in each row)

|  | **Above average (5)** | **Slightly above average (4)** | **Average (3)** | **Slightly below average (2)** | **Below average (1)** |
| --- | --- | --- | --- | --- | --- |
| Currently |  |  |  |  |  |
| While growing up |  |  |  |  |  |

4. How old were you when you began reading comics? ___________ Drawing comics? ____________

5. Are you familiar with the comic strip “Peanuts” by Charles Schulz (i.e., Snoopy and Charlie Brown)? (circle one) *Yes No*

6. How would you rate how much “Peanuts “ you have read? (check one)

|  | **Above average (5)** | **Slightly above average (4)** | **Average (3)** | **Slightly below average (2)** | **Below average (1)** |
| --- | --- | --- | --- | --- | --- |
| Currently |  |  |  |  |  |
| While growing up |  |  |  |  |  |
